# Assessment of Various Machine Learning Models for Peach Maturity Prediction Using Non-Destructive Sensor Data

**DOI:** 10.3390/s22155791

**Published:** 2022-08-03

**Authors:** Dejan Ljubobratović, Marko Vuković, Marija Brkić Bakarić, Tomislav Jemrić, Maja Matetić

**Affiliations:** 1 Faculty of Informatics and Digital Technologies, University of Rijeka, Radmile Matejčić 2, 51000 Rijeka, Croatia; dejan.ljubobratovic@uniri.hr (D.L.); mbrkic@uniri.hr (M.B.B.); majam@uniri.hr (M.M.); 2 Division of Horticulture and Landscape Architecture, Department of Pomology, Svetošimunska cesta 25, University of Zagreb Faculty of Agriculture, 10000 Zagreb, Croatia; mvukovic@agr.hr

**Keywords:** machine learning, AUC, peach maturity prediction, artificial neural networks, fruit quality, non-destructive measurements, dimensionality reduction, lasso regularization, group lasso

## Abstract

To date, many machine learning models have been used for peach maturity prediction using non-destructive data, but no performance comparison of the models on these datasets has been conducted. In this study, eight machine learning models were trained on a dataset containing data from 180 ‘Suncrest’ peaches. Before the models were trained, the dataset was subjected to dimensionality reduction using the least absolute shrinkage and selection operator (LASSO) regularization, and 8 input variables (out of 29) were chosen. At the same time, a subgroup consisting of the peach ground color measurements was singled out by dividing the set of variables into three subgroups and by using group LASSO regularization. This type of variable subgroup selection provided valuable information on the contribution of specific groups of peach traits to the maturity prediction. The area under the receiver operating characteristic curve (AUC) values of the selected models were compared, and the artificial neural network (ANN) model achieved the best performance, with an average AUC of 0.782. The second-best machine learning model was linear discriminant analysis with an AUC of 0.766, followed by logistic regression, gradient boosting machine, random forest, support vector machines, a classification and regression trees model, and k-nearest neighbors. Although the primary parameter used to determine the performance of the model was AUC, accuracy, F1 score, and kappa served as control parameters and ultimately confirmed the obtained results. By outperforming other models, ANN proved to be the most accurate model for peach maturity prediction on the given dataset.

## 1. Introduction

In the peach (*Prunus persica* (L.) Batsch) industry, proper fruit maturity determination at harvest is of prime importance for proper post-harvest manipulation [1,2,3], ensuring quality and consumer satisfaction [1,2,4,5]. Peaches are classified as climacteric fruits, characterized by a rapid increase in ethylene emission and respiration at the onset of ripening, accompanied by color, texture, aroma, and other biochemical changes [6]. Firmness, soluble solids concentration (SSC), and ground color changes are usually the most important methods used by producers for the determination of the harvest date with respect to appropriate maturity. Peach fruits ripen rapidly and have a short postharvest life, usually limited to 3–4 weeks depending on storage conditions [7]. Therefore, the supply chain is primarily focused on prolonging their storability to allow long distance export [8]. The post-harvest performance of peaches is mainly determined by flesh firmness [2,3]. Since melting peaches are very susceptible to rapid flesh firmness decline during the end of ripening and in the postharvest period [6,9], they are usually harvested at early maturity stages. There is a close link between “on-tree physiological maturity” and the evolution of key traits responsible for peach quality during the postharvest phase [1]. Fruits harvested at an unripe stage are more prone to shriveling, internal breakdown, and mechanical damage, and they are of inferior quality when ripe [4]. Consequently, the most important peach producing countries in Europe have lost considerable market shares mainly due to excessive early harvesting [1], while bad organoleptic attributes [5] are the main reasons why consumers do not eat more stone fruit.

The determination of peach maturity using destructive measurements is a slow process that results in fruit destruction, and it is usually conducted only on a certain (smaller) number of fruits, which can affect accuracy. On the other hand, manual sorting according to maturity fruit stage is tedious and time-consuming, and susceptible to discrepancies and inaccuracies if fruits are sorted by different human experts. In the light of that, peach maturity prediction based on sensory non-destructive data would present a notable improvement of various processes. Consequently, fruit maturity could be assessed.

Sensors could be used on a factory line to provide retailers with “up to date” information of a general peach maturity stage. This would help retailers to determine the further course of action in a more precise manner, e.g., penalize the producer with insufficiently ripe peaches, or return the shipment. In order to achieve this, machine learning models are used for data processing.

The application of machine learning to sensory data has already been successfully applied in agriculture. Such management systems, based on machine learning models, provide farmers with real-time recommendations and insights to assist them in the decision-making process [10]. One of the most common applications of machine learning in agriculture is the prediction of fruit maturity. To date, many studies have been conducted to predict fruit maturity using various machine learning models, and machine learning implementation in agriculture has been extensively researched. A random forest (RF) algorithm in combination with explainable machine learning methods was used by Ljubobratović et al. [11] to develop a machine learning model that identifies the most important features for predicting the maturity of peaches to detect nonlinear (and linear) relationships between them. In their study, Scalisi et al. [12] used partial least square (PLS) regression and linear discriminant analysis (LDA) algorithms for peach maturity prediction in different configurations of the spectrometer (fluorescence, near infrared spectroscopy (NIR), and RGB color model). In a study conducted by Sohaib et al. [13], spectral information was used to develop an NIR-based maturity estimator of various fruits (apple, mango, grapes, peaches, pears, and melons) using least squares support vector machine learning techniques. The RF machine learning algorithm was used by Ljubobratović et al. [14] for the prediction of ‘Spring Belle’ peach maturity, while RF and KNN models were successfully established to predict the maturity of peaches during shelf-life in another study [15]. Voss et al. [16] used three machine learning models, i.e., extreme learning machine, KNN, and support vector machines (SVM), for the prediction of peach fruit growth and maturation based on data collected using the E-nose prototype. Artificial neural network (ANN) models were used for fruit maturity prediction and classification in several studies [17,18,19]. However, up to our knowledge, a more detailed analysis of the fruit ripening prediction models and a comparison of their performance in this area has not yet been made. Thus, the aim of this study is to determine the best machine learning model for predicting the maturity of fruits, i.e., in this particular case, predicting the maturity of peaches.

Researchers often encounter a high dimensionality of the dataset, i.e., a large number of predictors, in their studies. To predict the maturity of fruits, Brezmes et al. [20] used the outputs of a large number of electronic nose sensors and then used principal component analysis (PCA) for reducing the dimensionality of results. The PCA method has also been used by Rajkumar et al. [21] to test the variability of the observed data in the studies related to banana fruit quality and maturity stages by using hyperspectral imaging.

Although the PCA method has already been used to reduce the dimensionality of datasets in predicting fruit maturity, one of the main disadvantages of this method is that the learned projective axes are actually linear combinations of all the original features. In this way, it is difficult to give a reasonable interpretation of which features play an important role in prediction [22]. The dataset used in this study included 30 nondestructive measurements on 180 peaches. The measurements are mainly related to peach dimensions and various color indices (Appendix A). Due to the large number of variables, dimensionality reduction was applied to the dataset to remove irrelevant features, as irrelevant features in the data can reduce the accuracy of the model by introducing model overfitting and cause the model to learn based on irrelevant features. To reduce the dimensionality of the dataset, least absolute shrinkage and selection operator method (LASSO) is used.

The group LASSO regularization method, which selects a subset of variables, was also used, and served not as a tool to reduce the dimensionality of the dataset, but as an indicator of certain peaches properties essential for the accurate prediction of peach maturity. Measurements of the ground color of peaches have been shown to have the greatest influence on the prediction of its maturity. Although this is not related to the selection of the best model directly, it helps in understanding the results and indicates a possible direction of future research.

Later, eight machine learning models were trained on the dimensionality reduced set of sensory data in order to predict the maturity of peach. The performance of the proposed models was compared and the model that gave the best results in predicting the maturity of peaches on the given dataset was selected. The models trained and compared in this study are: LDA, logistic regression (LR), classification and regression trees (CART), KNN, SVM, RF, gradient boosting machine (GBM), and ANN.

Therefore, the main objective of this study was to identify a machine learning model from the proposed models that has the best performance in predicting peach maturity using a set of non-destructive input parameters.

The later sections are organized as follows. The methods for measuring peaches and the description of the measured properties are described in Section 2.1. Section 2.2 describes the dataset and the procedure used to reduce its dimensionality (LASSO and group LASSO). Section 2.3 describes the machine learning models and the methods used to select the best model. The results are explained in the third section, and the best model, i.e., ANN, is presented. In the same section, the results are compared to those obtained with a dataset without dimensionality reduction. Section 4 and Section 5 contain a discussion and a conclusion. A complete list of the measured variables can be found in Appendix A.

## 2. Materials and Methods

‘Suncrest’ peaches of different maturity stages were harvested at the onset of August in a commercial orchard located near the city of Čakovec (Northern part of Croatia). In total, 180 peach fruits were harvested. The peaches were raised as an open vase on vineyard peach used as rootstock. The spacing was 4 m between peach rows and 3 m within rows. In the orchard, standard agro- and pomo-tehnical measures were regularly applied. ‘Suncrest’ peach is a late-maturing variety [23] originating from the USA (CA, USA) [24]. When ripe, it develops intense yellow (ground) and intense bright red skin colour (additional colour), while its flesh is yellow coloured. Additional colour overlays from 50 to 90% of its fruit surface [25].

### 2.1. Physico-Chemical Properties of Fruits

Immediately after the harvest, fruits were transferred to the laboratory of Department of Pomology at the Faculty of Agriculture of the University of Zagreb in Croatia, where all physicochemical analyses have been conducted.

#### 2.1.1. Ground (GC) and Additional (AC) Fruit Skin Color

On each fruit, ground and additional fruit skin color parameters were measured separately using a colorimeter (ColorTec PCM; ColorTec Associates Inc., Clinton, NJ, USA), according to the CIE L*a*b* and CIE L*C*h° systems (Commission Internationale d’eclairage).

The measurements with the colorimeter were made under laboratory conditions by using instruments and reading the displayed values. Since the measurement conditions were the same for all the samples, no preprocessing of the data was necessary.

In the CIE L*a*b* color space, the L* value corresponds to a dark-bright scale and represents the relative lightness of colors with a range from 0 to 100 (0 = black, 100 = white) [26]. The a* and b* scales extend from −60 to 60, where a* is negative for green and positive for red and b* is negative for blue and positive for yellow [26].

According to Carreño et al. [27], the hue angle (*h*°) and the chroma (C*) are calculated as given in Equations (1) and (2).

(1) $$h{}^{\circ}=\tan^{-1}\;\left(\frac{b*}{a*}\right)$$

(2) $$C={\left[{\left(a*\right)}^{2}+{\left(b*\right)}^{2}\right]}^{0.5}$$

where: a* and b*—variables in the CIE L*a*b system.

The hue angle (*h*°) describes the relative amounts of redness and yellowness, where 0°/360° is defined for red/magenta, 90° for yellow, 180° for green, and 270° for the blue color [28].

From the obtained color values, various ground and additional fruit color indexes were subsequently calculated:

(a) *a*/*b* color index.

The *a*/*b* ratio is used as a color index for tomatoes, citrus, red grapes, etc., [27,29,30,31]. It is calculated according to Equation (3).

(3) $$\frac{a}{b}=\frac{a^{*}}{b*}$$

where: a* and b*—variables in the CIE L*a*b system.

(b) Citrus color index (CCI).

The CCI color index is described by Jimenez-Cuesta et al. [32], and it is used for de-greening of citrus fruits. It is calculated according to Equation (4).

(4) $$CCI=\frac{1000\times a*}{L*\times b*}$$

where: L*, a*, and b*—variables in the CIE L*a*b* system.

(c) Tomato color index (COL).

The COL index, described by Hobson [33], is calculated by Equation (5).

(5) $$COL=\frac{2000\times a*}{L*\times c*}$$

where: L*, a* and C*—variables in the CIE L*a*b* and CIE L*C*h° systems.

(d) Red grape color index (CIRG^1^).

This index is designed by Carreño et al. [27] by modifying the index reported in [31]. It is calculated according to Equation (6).

(6) $$CIRG^{1}=\frac{180-h{}^{\circ}}{L*+C*}$$

where: L*, C*, and *h*°—variables in the CIE L*a*b* and CIE L*C*h° systems.

(e) Red grape color index (CIRG^2^).

This index is designed by Carreño et al. [27] by modifying the index reported in [31]. It is calculated according to Equation (7).

(7) $$CIRG^{2}=\frac{180-h{}^{\circ}}{L*\times C*}$$

where: L*, C*, and *h*°—variables in the CIE L*a*b* and CIE L*C*h° systems.

#### 2.1.2. Fruit Weight, Width, Length, Shape Index, Diameter, Volume, and Density

Fruit weight was measured using a digital analytical balance (OHAUS Adventurer AX2202, Ohaus Corporation Parsippany, Parsipanny, NJ, USA) with an accuracy of 0.01 g. Fruit length and width (mm) were measured with a digital scrolling scale Prowin HMTY0006 on two fruit sides. The fruit shape index was calculated by Equation (8).

(8) $$\mathrm{Fruit}\;\mathrm{shape}\;\mathrm{index}=\frac{\mathrm{fruit}\;\mathrm{length}}{\mathrm{fruit}\;\mathrm{width}}$$

Fruit radius was calculated as an average of fruit length and width values. Fruit volume was calculated by Equation (9).

(9) $$\mathrm{Fruit}\;\mathrm{volume}\;\left({\mathrm{cm}}^{3}\right)=\left(\frac{4}{3}\times \;\pi \right)\times {\;\mathrm{fruit}\;\mathrm{diameter}}^{3}\;$$

Fruit density was calculated according to Equation (10).

(10) $$\mathrm{Fruit}\;\mathrm{density}\;\left({g\;\mathrm{cm}}^{-3}\right)=\frac{\mathrm{fruit}\;\mathrm{mass}}{\mathrm{fruit}\;\mathrm{volume}}$$

### 2.2. Dataset and LASSO

The main goal of this study was to find the best machine learning peach maturity predicting model for a dataset with 180 observations. The original dataset included 30 nondestructive variables mainly related to peach morphological characteristics and measured by sensors (Appendix A).

According to minimal instrumental parameters, peaches at harvest should have firmness no more than 4.59 kg cm^−2^ ([7] according to [34]). Hence, in this study, this value was adopted as the firmness threshold. The output (predicting) variable was therefore binary variable *ripe* derived from peach firmness, representing peaches that have firmness no more than 4.59 kg cm^−2^.

In addition to the aforementioned *ripe* output variable, 7 of the remaining 29 variables in this dataset relate to peach weight, density, and dimensions, while the other 22 variables relate to peach ground and additional colors, as described in the previous section.

#### 2.2.1. LASSO

Measurements in this study included 30 variables, which is a lot in relation to the number of measurements. Too many variables can reduce the accuracy of the model and cause overfitting and learning based on irrelevant features [22]. Therefore, the LASSO method was used for feature subset selection in order to increase the accuracy of the models. LASSO is a machine learning technique for selecting a subset of relevant features or variables for constructing a model and eliminating redundant or irrelevant or highly correlated features without much loss of information [22]. LASSO was first introduced by Tibshirani [35] for parameter estimation and variable selection in regression analysis. It is a particular case of the penalized least squares regression with L1-penalty. LASSO, as a feature selection method, focuses on deleting irrelevant or redundant features as opposed to the PCA method that reduces dimensionality by combining features into a smaller number of new, derived features [36]. All calculations, predictions, visualizations, and the LASSO regularization coefficients were performed using the R programming language version 4.13 (R Foundation for Statistical Computing, Vienna, Austria) with the *caret*, *neuralnet*, *ggplot2*, and *glmnet* packages.

The LASSO regularization uses a modified least squares method in which the regression coefficients are calculated by minimizing the residual sum of squares increased by the sum of the absolute values of the coefficients multiplied by lambda (11) [22,37].

(11) $$RSS+\lambda \overset{p}{\underset{j=1}{\sum }}\left|\beta_{j}\right|$$

**Figure 1:**
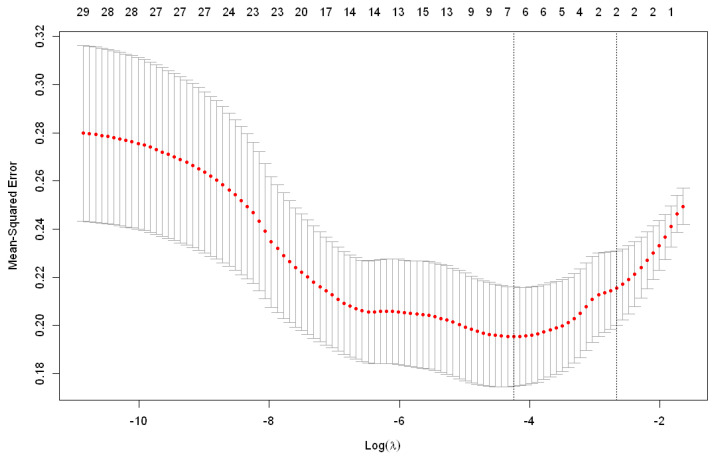
Graphical representation of MSE values obtained by cross-validation used to select the best lambda value.

Lambda is a tuning parameter and setting it to zero reduces the problem to the least squares method, while a sufficiently large value of lambda yields the null model, i.e., all regression coefficients are zero. The idea is to find a lambda that minimizes the mean squared error (MSE) for the mentioned function. For this purpose, a cross-validation is performed where the lambda value varies between 10^10^ and 10^−2^. In this study, the lambda for which MSE was lowest was 0.014 (Figure 1).

Using this method and the selected lambda value, 21 input variables were eliminated from the original dataset, leaving only 8 input variables for model training (Table 1), and predicting the output variable *ripe*.

#### 2.2.2. Dimension Reduction Using Group LASSO

The LASSO regularization reduces the number of variables in the dataset and retains only those variables that most contribute to the accurate prediction of the output variable.

There is also a regularization that selects specific groups of variables called the group LASSO. First introduced by Yuan et al. [38], the group LASSO is a type of the LASSO regularization for performing variable selection on predetermined groups of variables. In this study, this type of variable subgroup selection provided valuable information on the contribution of specific groups of peach properties to the prediction of maturity.

The data used in this study that consists of 29 variables were divided into 3 subgroups, with the first 7 variables forming Group 1 (morphological properties), the next 11 variables forming Group 2 (ground color properties), and the remaining variables forming Group 3 (additional color properties). List of variables and subgroups are shown in Table A1.

In the same way as for the LASSO regularization, the cross-validation (from the R package *gglasso*) was used to find the tuning parameter lambda for which MSE is the smallest. The group LASSO regularization was performed using the obtained lambda and it was found that Group 3 (ground color) is the most important for correctly predicting peach maturity. The results of the group LASSO regularization are shown in Table 2.

### 2.3. Training the Machine Learning Models

Eight different machine learning models trained on 8 input variables obtained by the LASSO regularization were used to predict the maturity of peaches. The output (predicting) variable was the derived binary variable *ripe* with a value of 1 for the measurements where the firmness of the peach was ≤4.59 kg cm^−2^ and a value of 0 for the measurements where it was not. The R packages used for prediction computations and visualizations were *caret*, *neuralnet*, and *ggplot2*. All models were trained on an HP ProDesk 600 computer with an i7 (3.2 GHz) processor, 16 GB RAM, and an Intel UHD 630 graphics card.

Before training, the dataset was divided into a training set containing 75% of the data and a test set containing the remaining 25% of the data. Thus, the training set contained 135 measurements and the test set contained 45 measurements.

As a training control mechanism, 7-fold cross validation repeated 3 times was used. Cross-validation is a statistical method for evaluating a learning algorithm in which the data are split into two segments, one used for learning and the other for validation [39]. The basic form of cross-validation is *k*-fold cross-validation. In *k*-fold cross-validation, the dataset is divided into *k* subsets of equal size, one of which is excluded for validation, while the other *k*−1 subsets are used for model training. Next another subset is taken for validation, while training is done with all the other subsets. After *k* iterations, each subset was used exactly once as a validation set and the performance of each iteration was determined. Various methods such as averaging can be used to obtain a cumulative result based on the performance of all iterations [39].

Since the training set contained 135 measurements, 10-fold cross-validation would test on subsets with 13 or 14 items, thus yielding less reliable results than 7-fold cross-validation performed on subsets with 19 or 20 measurements.

The selected machine learning models were trained on the given dataset and their performances were compared. The area under the receiver operating characteristic curve (AUC), accuracy, F1 score, and kappa parameters of given machine learning models were compared to select the model with the best results.

Model accuracy is defined as the percentage of correct predictions for the test data.

The F1 score is defined as the harmonic mean of recall and precision [40] as shown in (12), where the best value of the F1 score is one, and zero represents the worst value. According to Sasaki et al. [41], the F1 score was first introduced at the Fourth Message Understanding Conference (MUC-4) in 1992 [40], and its name was derived from van Rijsbergen’s book as the definition of the “F-measure” [42].

(12) $$F1=\frac{2\times P\times R}{P+R}$$

Precision (*P*) is given in Formula (13), where TP represents true positives (correctly predicted positive outcome) and FP represents false positives (falsely predicted positive outcome). The calculation of recall is shown in Formula (14), where FN stands for false negatives (falsely predicted negative outcome). 

Kappa parameter (Cohen’s Kappa) is a measure of model reliability, and it is a useful evaluation metric. Kappa is calculated as given in (13).

(13) $$\mathrm{kappa}\;=\frac{\mathrm{total}\;\mathrm{accuracy}\;-\;\mathrm{random}\;\mathrm{accuracy}}{1-\;\mathrm{random}\;\mathrm{accuracy}}$$

It tries to correct the evaluation bias by considering the correct classification obtained by a random guess. Kappa is within the range [−1, +1], where values closer to one indicate a more precise model.

The area under the receiver operating characteristic (ROC) curve is a two-dimensional measure of classification efficiency. AUC is a scalar measure that shows one aspect of its performance [43]. According to Bradley et al. [44], AUC is one of the best methods for evaluating the performance of a model when a “singular” evaluation is required. The authors in [45] find it to be a better measure of model performance than accuracy. AUC is therefore used as the primary performance measure for the selected machine learning algorithms.

Due to the diversity, models of varying complexity were used:

- LR and LDA (simple linear models),
- KNN and CART (nonlinear models),
- SVM, RF, and GBM (complex nonlinear models), and
- ANN (deep learning model).

The first two models are simple linear models. The LR model is an extension of the linear regression model in which, instead of fitting the line, the logistic function is used to fit the results of a linear Equation between 0 and 1 [46]. LR is considered a linear model because the boundary of the decision it generates is linear, which can be used for classification purposes [46,47], as is the case in this study. In this study, the generalized linear model from the Caret R package was used to train the LR model.

LDA is linear model used for dimensionality reduction and classification [48]. The LDA technique reduces dimensionality by transforming features into lower-dimensional space, maximizing the ratio of variance between classes and variance within a class, and thus maximizing the distance between classes [49]. According to the same authors, predictions are made by estimating the probability that a new set of input data belongs to each class, where the output class is the one that has the highest probability.

KNN method is a simple classification and regression method that classifies an object by finding the *k* nearest training examples in a dataset and forms its neighborhood [50]. The output is a class determined by a plurality vote of its neighbors. The object is therefore assigned to the class that is the most common among its *k* nearest neighbors [51].

CART is a classification technique that creates decision trees from input data, which can then be used to classify new observations [52]. It can also be used for regression, but that was not the case in this study.

SVMs are classifiers that distinguish data objects from two categories, where each object is represented by an *n*-dimensional vector and belongs to only one of the two classes [53]. The linear classifier separates them by a hyperplane, so the SVM selects the hyperplane with the largest margin to maximize the separation of the two classes [53]. The margin is the sum of the shortest distance between the separating hyperplane and the closest object from the two categories. This classification is then applied when predicting “unseen” or test objects. 

RF is an ensemble machine learning model for classification and regression first introduced by Breiman in 2001 [54]. It creates a large number of decision trees by using bagging and randomness of features in the creation of each tree and resulting in an uncorrelated forest of trees whose overall prediction is more accurate than that of any individual tree [54].

GBM is also an ensemble machine learning model that sequentially fits new models to obtain a more accurate estimate of the response variable by converting weak learners (weak models) into strong learners (strong models) [55,56].

ANN is a machine learning model whose basic idea is to simulate the function of the human brain and its basic unit, a neuron [57]. According to Mohammadhassani et al. [57], as in the real world, the ANN model consists of many neurons, each of which generates a set of activations with real value. Although similar, the idea of ANN is not to replicate the work of biological systems, but to use what is known about how biological networks work to solve complex problems [58].

The way ANN works is that the artificial neuron sums the weighted inputs and passes the result to the transfer function to produce the output [58]. This output is then sent to another neuron as input or used directly as a result of the network. Some inputs may be more important than others, and therefore, weights are used that correspond to the importance of each input and provide an effective way to generate ideal outputs. 

All models used the 7-fold cross-validation technique repeated three times. To ensure that each algorithm was evaluated using the same data divisions, the value of the random seed number was initially set to the same value before running the models. This resulted in model accuracies that varied widely depending on the value of the seed variable, partly due to the size of the test set. Table 3 shows the AUC results of all models when the seed changes from 1 to 5. The differences are large, e.g., greater than 20% for some models.

To avoid this variability, the models were trained with the seed values set from 1 to 100, and their averages were taken as the performance measure of each model. Thus, the fact that the models were trained 100 times with seven-fold cross-validation repeated three times, making a total of 16,800 training sessions, gives us confidence that the results are not accidental or biased.

After calculating the average AUC, accuracy, F1 score, and kappa value of each model, the one that came closest to these averages was selected, and this model was then used as a representative model.

## 3. Results

After training the individual models and comparing the average AUC, accuracy, F1 score, and kappa values, the ANN model proved to be the best model with the highest AUC (0.782), accuracy (0.738), F1 score (0.765), and kappa coefficient (0.468), followed by the LDA model with an AUC 0.766, accuracy of 0.730, and F1 score (0.765). The KNN model had the weakest performance with the lowest AUC (0.626), accuracy (0.605), and F1 score (0.653), while the other models AUCs ranged from 0.670 to 0.765. Table 4 shows the averaged AUC, accuracy, F1 score, and kappa values from 100 model trainings with different seed values, sorted by AUC.

**Figure 2:**
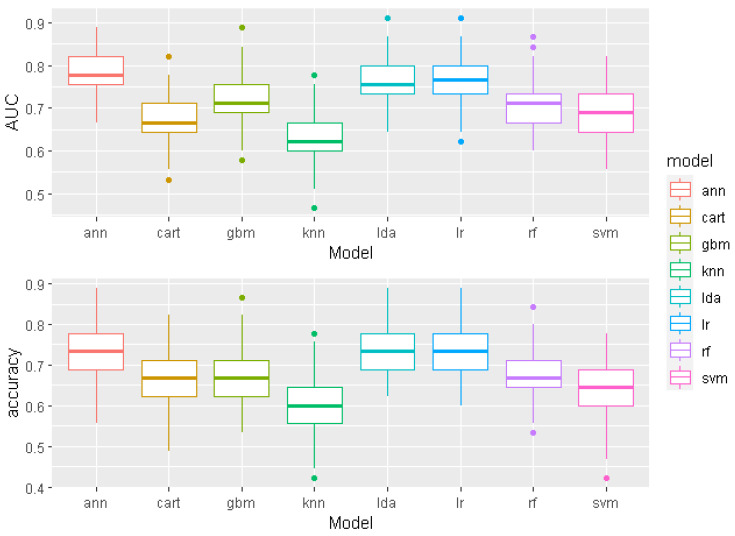
Comparison of AUC and accuracies for all eight models from 100 model trainings with different seed values.

The predictive models average AUC and accuracy boxplot comparisons are shown in Figure 2. The graphs show how much the model results vary for different seed values. For example, the KNN model, which proved to be the weakest on average, gave an AUC value of 0.778 and an accuracy of 77.8% for the seed value 56, which can clearly be seen as an anomaly in the graph. In this way, without considering other seed values, one could incorrectly conclude that the KNN model works well on that particular dataset.

**Figure 3:**
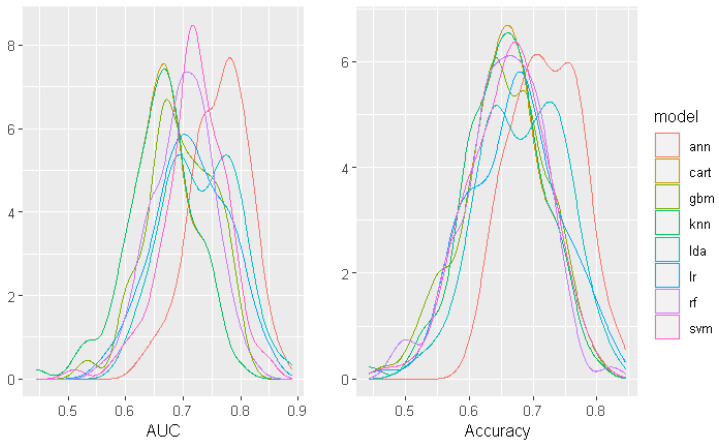
AUC and accuracy density distributions of compared models.

The density curves of AUC and accuracy parameters of the trained models are shown in Figure 3. The density curves of the ANN model are evidently left-skewed and the narrowest due to the highest scores, which means that ANN performs consistently well for most seed values.

### 3.1. Representative Models

The results given in Table 4 are average results per model obtained by 100 training runs with different seed values. In order to plot ROC curves and analyze individual models, for each model a seed that gave the most similar results to the average result was determined. Thus, for the average AUC, accuracy, F1 score, and kappa of each model, a corresponding representative model was found. Because the testing set contained only 45 measurements, the AUC and accuracies of the representative models changed in increments of 0.022. Therefore, for example, the AUC of a representative ANN model was reported as 0.778, although its average value was higher (0.782).

Table 5 shows average score values for individual models and the results of the corresponding representative models for the chosen seed values that most closely match these values.

**Figure 4:**
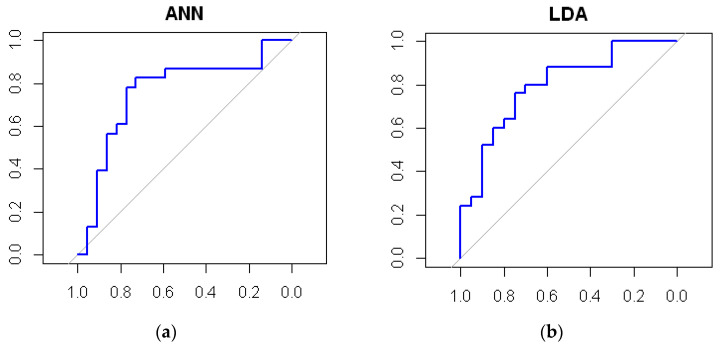

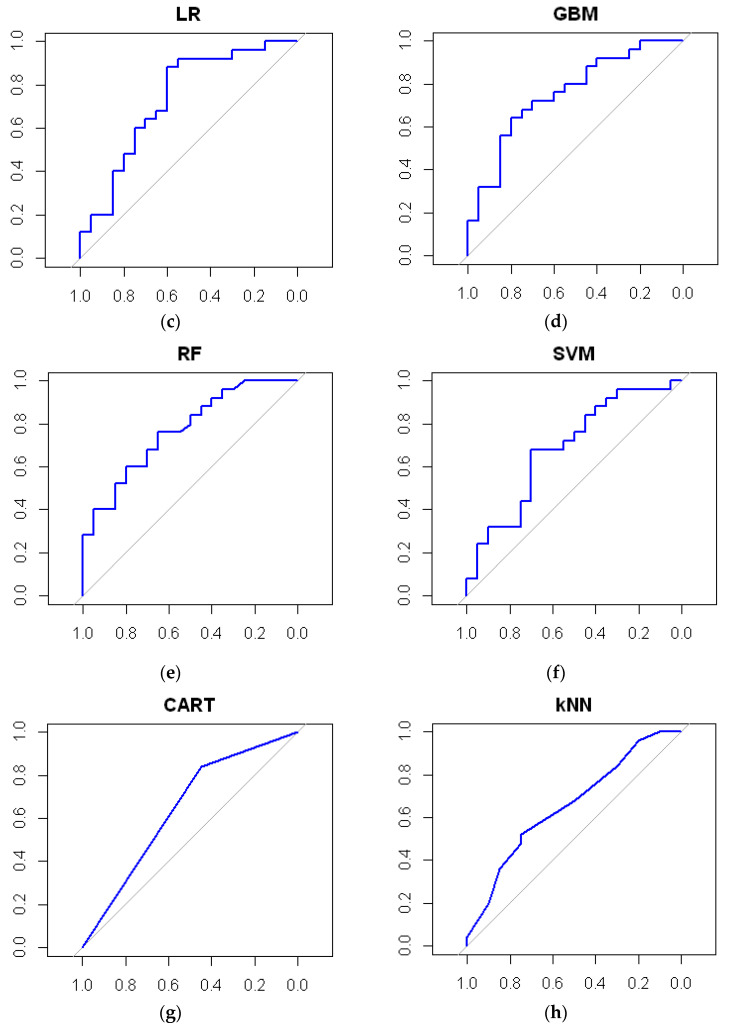
ROC curves for representing models.

Based on the results of the representative models, ROC curves were generated for each model (Figure 4). If AUC is approximately 0.5, it means that the model has no discrimination ability, and it is represented by a straight diagonal line. On the other hand, the maximum value for AUC is 1.0, indicating a theoretically perfect model [59].

It is noticeable that the curves of the CART and KNN models are “flatter” and closer to the diagonal, unlike those of the ANN or LDA models that form a larger arc and thus give a larger area underneath, i.e., a larger AUC.

### 3.2. The Best Model—ANN

**Figure 5:**
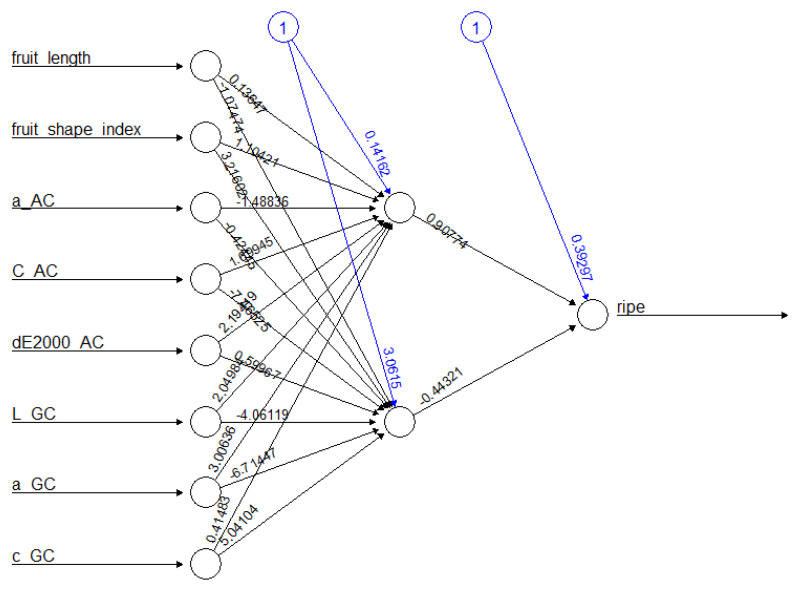
Representation of the ANN network with 8 input variables and 2 hidden layers with the output variable *ripe*.

This model had an average AUC of 0.782, an accuracy of 73.8%, an F1 score of 0.765, and a corresponding kappa of 0.468 (Table 4). The model that best represents these average results is the representative ANN model with two hidden layers (Figure 5).

### 3.3. Training the Model on the Entire Dataset

To justify the use of regularization, the predictions of the three models with the best performance were trained on all the available features of the dataset. The machine learning model results trained on the dataset with all 29 input variables compared to the results for the same algorithms trained on the lasso-reduced dataset are shown in Table 6.

All three models showed an increase in performance in all three measured parameters, with the largest increase in LR, where the obtained AUC is 7.14% better when using the LASSO regularization. 

**Figure 6:**
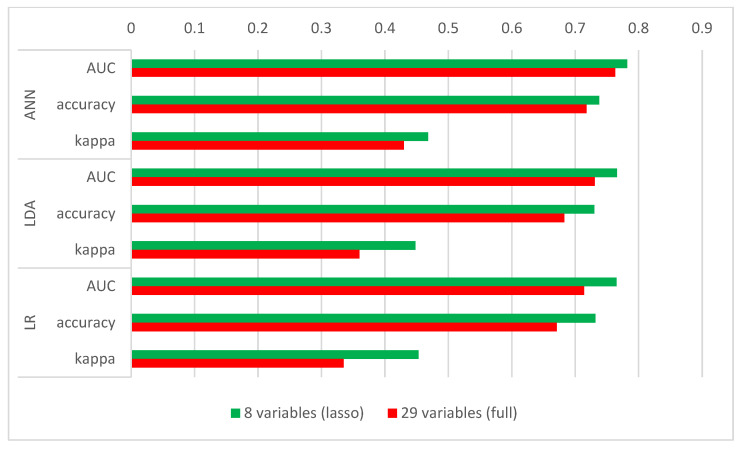
The graphical comparison of model performance shows an increase in all measured parameters for the models trained on the dataset to which LASSO was applied.

Figure 6 shows a graphical comparison of the performances of the best learning models trained on the full dataset and on the dataset reduced with the LASSO regularization.

## 4. Discussion

After training several models and comparing their average parameters, in this study, the model with the highest AUC, accuracy, F1 score, and kappa coefficient was determined. Although four parameters were considered (AUC, accuracy, F1 score, and kappa), AUC was used as the primary comparison parameter for all eight machine learning algorithms, since it is a better measure of model performance, as reported by Ling et al. [45]. A similar study was conducted by Bradley [44] comparing six machine learning algorithms with six sets of “real-world” medical diagnostic data in order to determine the one with the best performance. In the aforementioned study, AUC proves to be one of the best methods to evaluate the performance of a model on a dataset when a “single number” evaluation is required. 

Although the original dataset contained 30 variables, using the LASSO regularization, only eight features were selected to be used for training the models. The main features used for model training were fruit length, fruit shape index, a*-AC, C*-AC, dE2000-AC, L*-GC, a*-GC, and C*-GC. These features are important fruit characteristics that are genetically controlled and thus cultivar-specific (fruit length, fruit shape index) [60] or reflect important characteristics that are highly correlated to fruit maturity (color parameters) [4]. On the other hand, group LASSO found that Group 3 (ground color) was the most important for the correct prediction of peach maturity. This was expected since peach skin ground color is an important maturity prediction tool as it changes along with other important parameters (soluble solids, flesh firmness, and volatile compounds) [2,7]. According to Nascimento Nunes [61], the development of peach blush color is related to the light exposure rather than to the fruit maturation. The fact that peaches can be harvested from different canopy positions and orchards with or without applied nets (different light growing conditions), as indicated in the previous study by Ljubobratović et al. [14], explains why Group 2 (additional color) was not the most important for the correct prediction of peach maturity.

It has been shown that the results of the experiments vary greatly depending on the seed value, and a solution to this problem has been proposed. Models were trained with seed values set from 1 to 100, and average results were taken as the performance of each model.

In many statistical programs, random numbers are “calculated” using the so-called pseudo-random number generators—a recursive method that starts from the initial value determined by an input number called the “seed”. The random number generator in R (the statistical language used in this study) is based on the Mersenne Twister algorithm MT19937 [62]. Using the same seed makes it possible to reproduce the same results in calculations with a random element, e.g., randomly selecting elements from a set. Due to the small size of the dataset, the performance of the models varied widely for different seed values. For example, the AUC value of the CART model varied from 0.489 to 0.822, as shown in Figure 2. Therefore, the use of the mentioned method gave reliable average results that were not random or biased.

In this study, the model with the best results was the ANN model. This model had an average AUC of 0.782, an accuracy of 73.8%, an F1 score of 0.765, and a corresponding kappa of 0.468. The ANN model was also the most complex model in this study. The second-best model (LDA) was a linear model with an average AUC of 0.766. Although this model was one of the simplest, it provided the second-best result in this study. The simplest model in this study, LR, ranked very high in the table with an average AUC of 0.765, slightly less than LDA. The SVM, RF, and GBM models are complex nonlinear models but yielded intermediate results. Although more complex, they did not produce better results than the much simpler LR model. The GBM model was one of the most complex in the comparison, but its accuracy was not the best. This model was obviously not a good fit for the small dataset used in this study. The RF algorithm had an average model AUC of 0.708, placing it in the lower half of our model accuracy table. CART was the second to the worst model at the bottom of the table, i.e., to the KNN model, which gave the worst results in this study with an average AUC of 0.626. All these results are shown in Table 4. The fact that the accuracy, F1 score, and kappa values gave almost identical results for the model ranking confirms the justification of using the AUC parameter as the primary criterion for comparison. Moreover, the ANN model achieved the best results for all three measured parameters. 

An ANNs AUC value of 0.782 represents an “acceptable” predictive result, according to Hosmer et al. [47], but to achieve “excellent” or “outstanding” results, it is necessary to add new input parameters or to increase the dataset. In this study, only non-destructive variables were used for peach maturity prediction, given the fact that they have the possibility of being implemented in post-harvest processes with minimal deceleration.

The implementation of these non-destructive measurements in post-harvest processes could be achieved by adding sensors. To automate and speed up the data collection process, color computer vision with ANN could be used to detect the ground color from an image taken with an industrial camera. A similar method was used by Patel et al. [63] to detect bloodstains or dirt stains on poultry eggs. In a study conducted by Jiang et al. [64], neural networks were used to detect five common apple leaf diseases. With the development of convolutional neural networks, visual recognition, such as image classification, localization, and detection has led to excellent performance [65] and would be very suitable for peach ground color recognition. However, this could be applied only to peach varieties that have ground color (as the one in this study), while new non-destructive measurements should be studied for peach varieties with extremely little or no ground color.

## 5. Conclusions

The comparison of machine learning model training results showed that the ANN model had the best predictive performance. AUCs ranged from the weakest KNN model at the value of 0.626 to 0.782 for the best ANN model. The values of the accuracy, F1 score, and kappa parameters were also considered, producing almost identical ranking results.

In this work, the primary concern was not only to predict fruit maturity, but to find the model that gives the best results on the given dataset, which consisted mainly of the color measurements of peaches. The original dataset contained 29 input variables, and the LASSO regularization method reduced their number to only eight. The measurement showed that this method, in addition to reducing the dimensionality of the set, simultaneously increased the accuracy of the model by more than 2% for the best model, i.e., ANN, and even more for the other models. Since the LASSO regularization proved useful, the group LASSO method was also used. The group containing the measurements of the ground color was selected as the most relevant for the successful prediction of peach maturity from the three pre-determined subsets of variables.

However, including measurements of other non-destructive parameters, such as peach electrical impedance, NIR, spectroscopy, or ‘electrical nose’ might give much better results. In our future research, we will therefore strive to increase the dataset and include other non-destructive parameters.

## Figures and Tables

**Table 1:** A dataset with the list of variables used in model training.

Feature	Variable Name	Description
fruit maturity	ripe	peach maturity (output binary variable)
fruit length	fruit_length	peach length
fruit shape index	fruit_shape_index	peach shape index
a*-AC	a_AC	a* variable of additional fruit color
C*-AC	C_AC	C* variable of additional fruit color
dE2000-AC	dE2000_AC	dE2000 for additional color
L*-GC	L_GC	L* variable of ground fruit color
a*-GC	a_GC	a* variable of ground fruit color
C*-GC	c_GC	C* variable of ground fruit color

**Table 2:** Group LASSO regularization preserving the coefficients next to the variables in Group 3.

Variable	Group	Group Lasso
fruit_weight	1	0.000000000
fruit_width	1	0.000000000
fruit_length	1	0.000000000
fruit_shape_index	1	0.000000000
fruit_diameter	1	0.000000000
fruit_volume	1	0.000000000
fruit_density	1	0.000000000
L_AC	2	0.000000000
a_AC	2	0.000000000
b_AC	2	0.000000000
C_AC	2	0.000000000
h_AC	2	0.000000000
a.b_AC	2	0.000000000
CCI_AC	2	0.000000000
COL_AC	2	0.000000000
CIRG1_AC	2	0.000000000
CIRG2_AC	2	0.000000000
dE2000_AC	2	0.000000000
L_GC	3	−0.003395380
a_GC	3	0.029737581
b_GC	3	0.005994080
c_GC	3	0.014852482
h_GC	3	−0.025684634
a.b_GC	3	0.024926216
CCI_GC	3	0.022079283
COL_GC	3	0.022994860
CIRG1_GC	3	0.011642995
CIRG2_GC	3	0.004742768
dE2000_GC	3	0.008545801

**Table 3:** Different models AUC results for seed values from 1 to 5.

Seed	ANN	CART	GBM	LDA	LR	KNN	RF	SVM
1	0.756	0.756	0.822	0.844	0.867	0.600	0.800	0.778
2	0.778	0.667	0.756	0.756	0.733	0.644	0.711	0.733
3	0.711	0.711	0.733	0.756	0.756	0.689	0.733	0.689
4	0.844	0.756	0.756	0.844	0.844	0.644	0.667	0.756
5	0.800	0.600	0.756	0.689	0.689	0.578	0.711	0.644

**Table 4:** Model’s AUC, accuracy, F1 score, and kappa averages.

Model	AUC	Accuracy	F1 Score	Kappa
ANN	0.782	0.738	0.765	0.468
LDA	0.766	0.730	0.765	0.448
LR	0.765	0.732	0.765	0.453
GBM	0.714	0.675	0.724	0.333
RF	0.708	0.675	0.722	0.332
SVM	0.691	0.642	0.688	0.267
CART	0.670	0.663	0.719	0.301
KNN	0.626	0.605	0.653	0.197

**Table 5:** Comparison of averaged model scores and representative model scores based on the chosen seed values.

Model	Representative Model Seed	Average AUC	Representative Model AUC	Average Accuracy	Representative Model Accuracy	Average Kappa	Representative Model Kappa
ANN	6	0.782	0.778	0.738	0.733	0.468	0.467
LDA	58	0.766	0.756	0.730	0.733	0.448	0.460
LR	3	0.765	0.756	0.732	0.733	0.453	0.449
GBM	29	0.714	0.711	0.675	0.667	0.333	0.322
RF	35	0.708	0.711	0.675	0.667	0.332	0.328
SVM	63	0.691	0.689	0.642	0.644	0.267	0.273
CART	18	0.670	0.667	0.663	0.667	0.301	0.301
KNN	29	0.626	0.622	0.605	0.600	0.197	0.182

**Table 6:** Results of the best performing models trained on the full dataset compared to the results of a model trained on the dataset with only 8 input variables (LASSO).

Model	AUC (Lasso)	AUC (Full)	AUC Increase	Acc. (Lasso)	Acc. (Full)	Acc. Increase	Kappa (Lasso)	Kappa (Full)	Kappa Increase
ANN	0.782	0.763	2.49%	0.738	0.718	2.79%	0.468	0.430	8.84%
LDA	0.766	0.731	4.79%	0.730	0.683	6.88%	0.448	0.360	24.4%
LR	0.765	0.714	7.14%	0.732	0.671	9.09%	0.453	0.335	35.2%

## Data Availability

Not applicable.

## Appendix A

**Table A1:** A dataset with the list of variables it contained before the dimension was reduced.

Feature	Variable Name	Description	Group
fruit firmness	firmness	peach firmness	output var.
fruit weight	fruit_weight	peach weight	1
fruit width	fruit_width	peach width	1
fruit length	fruit_length	peach length	1
fruit shape index	fruit_shape_index	peach shape index	1
fruit diameter	fruit_diameter	peach diameter	1
fruit volume	fruit_volume	peach volume	1
fruit density	fruit_density	peach density	1
*L**-AC	L_AC	*L** variable of additional fruit color	*2*
*a**-AC	a_AC	*a** variable of additional fruit color	*2*
*b**-AC	b_AC	*b** variable of additional fruit color	*2*
*C**-AC	C_AC	*C** variable of additional fruit color	*2*
*h*°-AC	h_AC	*h*° variable of additional fruit color	*2*
*a**/*b**-AC	a.b_AC	*a**/*b** additional color index	*2*
CCI-AC	CCI_AC	CCL additional color index	2
COL-AC	COL_AC	COL additional color index	2
CIRG^1^-AC	CIRG1_AC	CIRG^1^ additional color index	2
CIRG^2^-AC	CIRG2_AC	CIRG^2^ additional color index	2
dE2000-AC	dE2000_AC	dE2000 for additional color	2
*L**-GC	L_GC	*L** variable of ground fruit color	*3*
*a**-GC	a_GC	*a** variable of ground fruit color	*3*
*b**-GC	b_GC	*b** variable of ground fruit color	*3*
*C**-GC	c_GC	*C** variable of ground fruit color	*3*
*h*°-GC	h_GC	*h*° variable of ground fruit color	*3*
*a**/*b**-GC	a.b_GC	*a**/*b** ground color index	*3*
CCI-GC	CCI_GC	CCL ground color index	3
COL-GC	COL_GC	COL ground color index	3
CIRG^1^-GC	CIRG1_GC	CIRG^1^ ground color index	3
CIRG^2^-GC	CIRG2_GC	CIRG^2^ ground color index	3
dE2000-GC	dE2000_GC	dE2000 for ground color	3
